# Choroidal metastases in testicular choriocarcinoma, successful treatment with chemo- and radiotherapy: a case report

**DOI:** 10.1186/1471-2490-11-24

**Published:** 2011-12-07

**Authors:** Ivo Guber, Leonidas Zografos, Ann Schalenbourg

**Affiliations:** 1 Jules-Gonin Eye Hospital, University of Lausanne, Switzerland, Avenue de France 15, CH-1004 Lausanne, Switzerland

## Abstract

**Background:**

Choriocarcinoma is a very rare cause of ocular metastasis. Only 18 male patients have been reported on, 4 of whom survived, but with significant loss of vision.

**Case presentation:**

A 26-year-old Caucasian man, suffering from testicular choriocarcinoma with pulmonary, cerebral, renal, hepatic and osseous metastases, underwent left radical orchiectomy. While being treated with chemotherapy, he presented with loss of vision in the left eye. Ophthalmoscopy revealed bilateral non-pigmented, hemorrhagic choroidal tumours, compatible with secondary lesions. Continued chemotherapy and stereotactic radiotherapy of the skull and spine lead to full remission with excellent vision, after more than 4 years of follow up.

**Conclusion:**

Testicular choriocarcinoma is an exceptional cause of choroidal metastasis, potentially asymptomatic and with specific clinical features. Radiotherapy can complement radical orchiectomy and chemotherapy, to achieve full remission and maintain good vision.

## Background

Intraocular metastases are present in up to 10% of patients with systemic malignancies [1]. With tumour cells spreading hematogenously, the posterior choroid is frequently involved because of its rich vasculature [2]. In females, the most common primary site is the breast, whereas, in males, it is the lung.

Choriocarcinoma is a highly malignant metastatic tumour, derived from undifferentiated placental trophoblasts ('gestational choriocarcinoma') or from non-placental totipotent cells, present in the testis, the ovary or the pineal gland ('non gestational choriocarcinoma'). It is characterized by the large amounts of chorionic gonadotropin produced.

Choriocarcinoma is a rare cause of choroidal metastasis. Only 18 male patients have been reported on, and most of them had a poor prognosis [2-4]. We present a man who developed bilateral choroidal metastases from a testicular choriocarcinoma under chemotherapy. The ocular tumours regressed following irradiation. Four years later, he is in remission and has excellent vision.

## Case presentation

A 26-year-old man was referred to the Jules-Gonin Eye Hospital with loss of vision and a non-pigmented tumour in his left eye. In the previous two weeks, he had been diagnosed with a left testicular tumour and multiple metastases to the lung, brain, cerebellum, kidney, liver and spine. Histopathology following left radical orchiectomy had revealed a non-seminomatous testicular choriocarcinoma, positive for β-human chorionic gonadotropin (β-HCG) and pancytokeratin, slightly positive for α-fetoprotein (AFP) while negative for carcino-embryonic antigen (CEA). Serum levels were for β-HCG 271 052 U/l (normal < 3.0 U/l)), for AFP 0.9 ng/ml (normal < 10 ng/ml) and for LDH 1757 U/l (normal < 450 U/l). He was receiving chemotherapy with bleomycin, etoposide and cisplatin.

**Figure 1:**
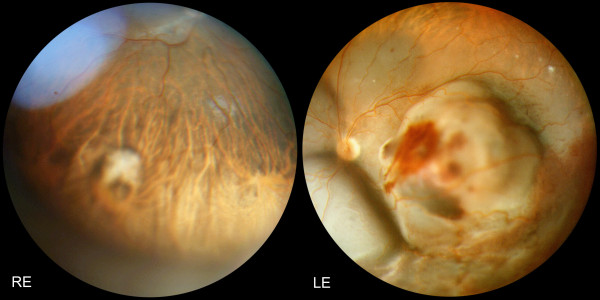
**Panoramic fundus pictures: Choroidal metastases from testicular choriocarcinoma at presentation: RE (Right Eye): a non pigmented, flat lesion is discovered in the inferior choroid**. LE (Left Eye): a large amelanotic tumour with associated hemorrhagic retinal detachment occupies the posterior pole and is responsible for the vision loss.

Visual acuity was 6/6 in the right eye and counting fingers (CF) in the left eye. The left posterior fundus showed a large amelanotic choroidal tumour associated with a hemorrhagic retinal detachment. On B-scan ultrasonography, thickness was 7 mm. In the right inferior choroid, a second non pigmented, flat lesion was also present, compatible with a diagnosis of bilateral choroidal metastases (Figure 1).

Chemotherapy was continued, with a total of 4 cycles over 3 months' time. Additionally, a stereotactic radiotherapy of the spine and head was performed, the latter including the posterior part of both eyes, using two lateral accelerated photon fields of 6 MV with a total dose of 30.80 Gy in 14 fractions.

**Figure 2:**
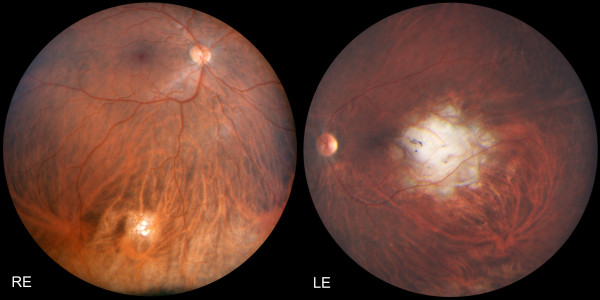
**Panoramic fundus pictures: Four years after chemo- and radiotherapy, both lesions have become flat chorioretinal scars**. Despite the unfavourable localisation of the left scar near the fovea, visual acuity has recovered to 6/9.

After four years' follow-up, the patient is in remission without any evidence of metastases. All tumour markers are within normal ranges (β-HCG < 0.1 U/l; AFP 1.2 ng/ml; LDH 284 U/l). Visual acuity is 6/6 in the right and 6/9 in the left eye, both fundi showing a flat chorioretinal scar (Figure 2).

## Discussion

We describe a rare case of a patient with bilateral choroidal metastases secondary to testicular choriocarcinoma, successfully treated with chemo- and radiotherapy.

MacDonald described the first case in 1936 [5]. A meta-analysis of the literature in 2006 listed 14 male patients with testicular choriocarcinoma, as well as one with mediastinal and one with retroperitoneal primary choriocarcinoma, metastasizing to the choroids [2]. Since then, 2 more cases of choroidal metastases from testicular choriocarcinoma have been reported [3,4].

Unlike other metastatic ocular disease, significant haemorrhage is associated with choriocarcinoma metastases in more than 85% of cases and should raise suspicion for this primary [2-4]. Kavanagh suggested a correlation with upregulated levels of vascular endothelial growth factor (VEGF) [2], thought to be involved in the development of trophoblastic disease [6].

Prognosis of testicular germ cell tumours -including choriocarcinoma- was poor until the introduction of cisplatin-based chemotherapy in the mid-1970s [3]. Of the 18 male choriocarcinoma patients with choroidal metastases, only 4 were reported to be cured, the first being described in 1999 [7]. Two received only chemotherapy [3,8], one had a supplementary brain radiotherapy excluding the orbit [7] and one had 'concurrent radiotherapy to the whole skull and left eye' [9], though no report specifies the radiation dosage. In our patient, the indication for stereotactic radiotherapy was based on the fact that visual symptoms appeared after chemotherapy had been initiated and that the blood-brain and blood-eye barrier are reputed to be only partially permeable to chemotherapeutics.

Visual acuity of the 4 surviving patients, who all had unilateral choroidal metastases, ranges between CF and 6/12 [3,7-9]. Our patient, with bilateral metastatic disease, has a visual acuity of 6/6 and 6/9 respectively, despite the parafoveal localization of the left tumour. The coincidentally discovered asymptomatic tumour in the right eye was treated before vision was affected.

## Conclusion

We describe a rare case of a patient with testicular choriocarcinoma presenting bilateral choroidal metastases, with specific clinical features and one eye being asymptomatic. Stereotactic radiotherapy of the skull and spine complemented radical orchiectomy and chemotherapy. After more than 4 years of follow up, he is in full remission and has excellent vision.

## Consent

Written informed consent was obtained from the patient for the publication of this case report and accompanying images. A copy of the written consent is available for review by the Editor-in-Chief of this journal.

## Competing interests

The authors declare that they have no competing interests.

## Authors' contributions

IG and AS drafted the manuscript. IG, LZ and AS cared for the patient. All authors reviewed the report and approved the final version of the manuscript.

## Pre-publication history

The pre-publication history for this paper can be accessed here:

http://www.biomedcentral.com/1471-2490/11/24/prepub
